# *CaWRKY40b* in Pepper Acts as a Negative Regulator in Response to *Ralstonia solanacearum* by Directly Modulating Defense Genes Including *CaWRKY40*

**DOI:** 10.3390/ijms19051403

**Published:** 2018-05-08

**Authors:** Muhammad Ifnan Khan, Yangwen Zhang, Zhiqin Liu, Jiong Hu, Cailing Liu, Sheng Yang, Ansar Hussain, Muhammad Furqan Ashraf, Ali Noman, Lei Shen, Xiaoqin Xia, Feng Yang, Deyi Guan, Shuilin He

**Affiliations:** 1 National Education Ministry, Key Laboratory of Plant Genetic Improvement and Comprehensive Utilization, Fujian Agriculture and Forestry University, Fuzhou 350002, China; mifnan@yahoo.com (M.I.K.); zhangzhang201711@163.com; (Y.Z.); lzqfujian@126.com (Z.L.); hjfujian@126.com (J.H.); lclfujian@126.com (C.L.); yangsheng2061@163.com (S.Y.); ahtraggar@yahoo.com (A.H.); furqan2210uaf@hotmail.com (M.F.A.); alinoman@gcuf.edu.pk (A.N.); shorttubelycoris07@163.com (L.S.); s1042657738@a163.com (X.X.); m17750292623@163.com (F.Y.); gdyfujian@126.com (D.G.); 2 College of Crop Science, Fujian Agriculture and Forestry University, Fuzhou 350002, China; 3 Key Laboratory of Applied Genetics of Universities in Fujian Province, Fujian Agriculture and Forestry University, Fuzhou 350002, China; 4 Department of Botany, Government College University, Faisalabad 38040, Pakistan

**Keywords:** *Capsicum annuum*, *Ralstonia solanacearum*, *CaWRKY40b*, immunity, negative regulator, transcriptional modulation

## Abstract

WRKY transcription factors (TFs) have been implicated in plant growth, development, and in response to environmental cues; however, the function of the majority of pepper WRKY TFs remains unclear. In the present study, we functionally characterized *CaWRKY40b*, a homolog of *AtWRKY40*, in pepper immunity. *Ralstonia solanacearum* inoculation (RSI) in pepper plants resulted in downregulation of *CaWRKY40b* transcript, and green fluorescent protein (GFP)-tagged CaWRKY40b was localized to the nuclei when transiently overexpressed in the leaves of *Nicotiana benthamiana*. Virus-induced gene silencing (VIGS) of *CaWRKY40b* significantly decreased pepper’ susceptibility to RSI. Consistently, the transient over-expression of *CaWRKY40b-SRDX* (chimeric repressor version of *CaWRKY40b*) triggered cell death, as indicated by darker trypan blue and DAB staining. CaWRKY40b targets a number of immunity-associated genes, including *CaWRKY40 JAR*, *RLK1*, *EIN3*, *FLS2*, *CNGIC8*, *CDPK13*, and *heat shock cognate protein 70* (*HSC70)*, which were identified by ChIP-seq and confirmed using ChIP-real time PCR. Among these target genes, the negative regulator HSC70 was upregulated by transient overexpression of *CaWRKY40b* and downregulated by silencing of *CaWRKY40b*, whereas other positive regulators as well as two non-target genes, *CaNPR1* and *CaDEF1*, were downregulated by the transient overexpression of *CaWRKY40b* and upregulated by *CaWRKY40b* silencing or transient overexpression of *CaWRKY40b-SRDX*. In addition, CaWRKY40b exhibited a positive feedback regulation at transcriptional level by directly targeting the promoter of itself. In conclusion, the findings of the present study suggest that *CaWRKY40b* acts as a negative regulator in pepper immunity against *R. solanacearum* by transcriptional modulation of a subset of immunity-associated genes; it also represses immunity in the absence of a pathogen, and derepresses immunity upon pathogen challenge.

## 1. Introduction

During the course of evolution, plants have developed a sophisticated defense mechanism for counteracting diverse pathogens. In addition to the physical barriers for damage prevention, plants possess two layers of inducible immunities termed as pathogen-associated molecular pattern (PAMP) triggered immunity (PTI) and effector triggered immunity (ETI). The perception of conserved microbe-associated molecular patterns (MAMPs) in plant triggers PTI via pattern recognition receptors (PRRs) on the plant cell surface. However, ETI in plants is triggered by the perception of strain-specific pathogen effectors that are delivered into host cells via intracellular R proteins, which are generally coupled with hypersensitive response (HR) cell death. Interconnected in a zig-zag manner [1,2,3,4,5,6], PTI and ETI share a highly overlapping signaling network [7,8,9]. A key step in both PTI and ETI is massive reprogramming that is mediated by various transcription factors (TFs) [10,11,12], which play vital roles in plant responses to the pathogen attack [13,14]. However, the roles of the majority of TFs in plant immunity and how these translate upstream stress signals into appropriate transcriptional outputs remain to be elucidated.

WRKYs constitute one of the largest plant TF families and have been implicated in plant growth and development as well as responses to various environmental stresses. These are named after their one or two WRKY domains, which comprise a highly conserved amino acid sequence WRKYGQK, together with a zinc-finger-like motif [15]. WRKY TFs have the ability to activate or repress transcription by directly binding to the W-box (TTGACC/T) within the promoters of their target genes [16], thereby playing important roles in the regulation of plant responses to pathogen and herbivore attacks as well as abiotic stresses, including heat stress, drought, and salinity. A subset of WRKYs have been found to be involved in plant immunity [17,18,19,20,21]. For example, among the 72 WRKY TFs in *Arabidopsis*, 49 *AtWRKY* genes are differentially expressed during the infection with *Pseudomonas syringae* or application of exogenous salicylic acid (SA) [22]. Genetic evidence demonstrates that *WRKY8* [23], *WRKY22* [24], *WRKY25* [25,26], *WRKY28* [23], *WRKY33* [25,27], *WRKY38* [28], *WRKY48* [23], *AtWRKY54* [29], *WRKY62* [28], *AtWRKY72* [30], and *WRKY75* [31] act as positive regulators, whereas *WRKY11*, *WRKY17* [32], *WRKY40* [33], and *WRKY60* [34] act as negative regulators in basal defense or immunity. WRKY TFs are part of a WRKY network and play important roles in the regulation of plant immunity [35,36]. However, the exact functions of other pathogen-responsive WRKY TFs and the modes of coordination among multiple WRKY TFs participating in plant immunity in non-model plant families such as the Solanaceae remain unclear.

Pepper (*Capsicum annuum*) is an important vegetable and a typical member of the Solanaceae. It is distributed in uplands during warm seasons and is constantly challenged with various soil-borne pathogens. The frequent occurrence of high temperature and high humidity (HTHH) conditions usually causes severe diseases in pepper plants. Breeding and the application of pepper cultivars that are highly resistant to disease is one of the most efficient approaches to solving the problem caused by diseases in pepper production, and a better understanding of the mechanism underlying pepper immunity against various pathogens may facilitate in the genetic improvement of pepper disease resistance. Although WRKY TFs play important roles in plant immunity, the number of WRKY TFs in pepper that have been characterized in terms of function and expression in relation to plant immunity is limited. They include: *CaWRKY1* [37], *CaWRKY-a* [38,39], *CaWRKY6* [3], *CaWRKYb* [40], *CaWRKYd* [41], *CaWRKY2* [42], *CaWRKY27* [43], *CaWRKY40* [4], and *CaWRKY58* [44]. In addition, some pepper WRKY TFs such as *CaWRKY6* [3] and *CaWRKY40* [4] also act as positive regulators of responses to high temperature stress, probably reflecting the evolution of special immunity under the combined pressure of HTHH and soil-borne pathogens. However, the exact function of the majority of the WRKY TFs in pepper remains elusive. In the present study, we focused on evaluating the function of *CaWRKY40b* in *R. solanacearum*-infected *C. annuum* plants. *CaWRKY40b* was found to act as a negative regulator in pepper immunity by directly targeting immunity-associated genes, including *CaWRKY40*.

## 2. Results

### 2.1. Sequence Analysis of CaWRKY40b

A pepper WRKY gene (*CA03g32070*) with unknown function previously identified by genome-wide analysis (http://passport.pepper.snu.ac.kr) was selected for functional characterization in relation to plant immunity, and a subset of immunity-associated *cis*-elements, including TGACG motif, TCA elements, TC-rich repeats, and W-box, was identified in its promoter region. The deduced amino acid sequence of *CA03g32070* was 360 amino acids in length, harboring one conserved WRKY domain. Among all members of the WRKY family in *Arabidopsis*, it shares highest sequence identity with *AtWRKY40*. We designated it as *CaWRKY40b* to distinguish from *CaWRKY40*, a positive regulator of pepper immunity to *Ralstonia.* [4]. *CaWRKY40b* shares 87%, 82%, 71%, 58%, and 45% amino acid sequence identity with its homologs in *S. lycopersicum* (XP_006341684.1), *S. tuberosum* (XP_006356251.1), *N. sylvestris* (XP_009802478.1), *A. thaliana* (NP_178199.1), and *O. sativa* (Q6IEK5.1), respectively, and 59.46% sequence identity to *CaWRKY40* [4]. According to the conserved WRKY domain and proposed structure of the zinc finger motif [16], *CaWRKY40b* belongs to Group IIa of the WRKY family-like *AtWRKY40* [16] and *CaWRKY40* [4] (Figure S1).

### 2.2. The Expression of CaWRKY40b Is Modulated Transcriptionally by Ralstonia solanacearum Inoculation (RSI)

**Figure 1:**
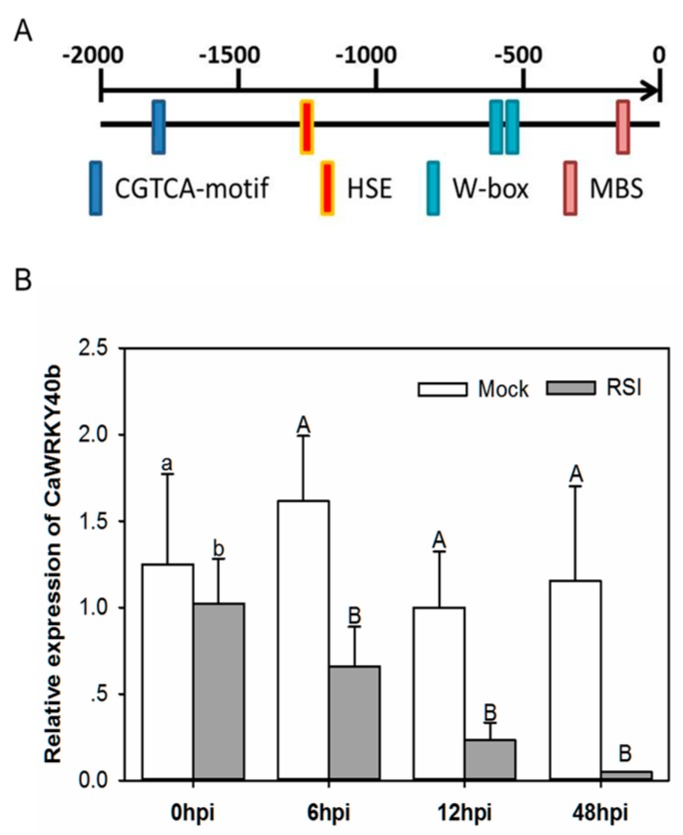
qRT-PCR analysis of relative *CaWRKY40b* transcript levels in *Ralstonia solanacearum*-inoculated pepper leaves. (**A**) Defense associated *cis*-element CGTCA-motif and W-boxes were detected in the promoter region of *CaWRKY40b* by plant care (http://bioinformatics.psb.ugent.be/webtools/plantcare/html/). (**B**) *CaWRKY40b* transcript levels measured at different time points in *R. solanacearum*-inoculated leaves after inoculation with virulent *R. solanacearum* strain FJC100301 (OD600nm = 0.8) were compared to that in control plants at relative expression level of “1′. Experiments were performed thrice in triplicate biological repeats. Data are expressed as the mean ± SD of three samples, each containing three plants. Different letters indicate significant differences as determined by Fisher’s protected LSD test: uppercase letters, *p* < 0.01; lower case letters, *p* < 0.05.

The presence of the subset of pathogen-responsive CGTGA motif [45] and W-box bound by WRKY TFs [16,17] in the *CaWRKY40b* promoter region implies its possible transcriptional response to pathogens. To test this possibility, the expression of *CaWRKY40b* in pepper plants against *R. solanacearum* was assessed by qRT-PCR. The results revealed a downregulation of *CaWRKY40b* by RSI in the pepper leaves from 6 hours post inoculation (hpi) to 48 hpi (Figure 1), indicating that *CaWRKY40b* might be involved in the response of pepper to RSI.

### 2.3. CaWRKY40b Localizes Exclusively to the Nuclei

**Figure 2:**
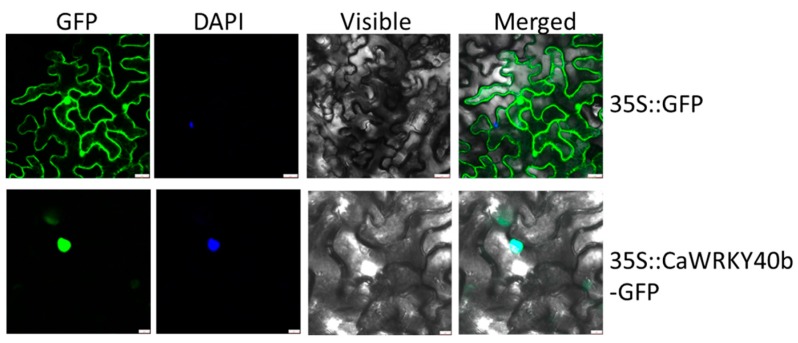
Subcellular localization of *CaWRKY40b.* The leaves of 50-day-old *Nicotiana benthamiana* plants were infiltrated with *Agrobacterium* strain GV3101 cells containing the *35S:CaWRKY40b-GFP* and *35S:GFP* construct, respectively. After 48 hpi, GFP fluorescence was imaged under a confocal microscope. The GFP signals in leaves infiltrated with GV3101 cells containing the *35S:CaWRKY40b-GFP* were observed in the nuclei, while that in leaves infiltrated with GV3101 cells containing *35S:GFP* construct were observed throughout the cell. DAPI, 4′,6-diamidino-2-phenylindole; GFP, green fluorescent protein, bar = 25 µm.

To localize *CaWRKY40b* at subcellular level, a fused *CaWRKY40b-GFP* protein was expressed in *N. benthamiana* leaves by infiltrating GV3101 cells containing *35S::CaWRKY40b-GFP* and using *35S::GFP* construct as a control. The GFP signal was observed under a laser scanning confocal microscope (LSCM). In *CaWRKY40b-GFP* transiently overexpressed epidermal cells of *N. benthamiana* leaves, the GFP signal was exclusively observed in the nuclei, whereas the GFP signals in the epidermal cells of *35S::GFP*-infiltrated *N. benthamiana* leaves were found across the entire cell, including plasma membrane, cytosol, and nucleus (Figure 2).

### 2.4. Silencing of CaWRKY40b Enhances the Resistance of Pepper to RSI

**Figure 3:**
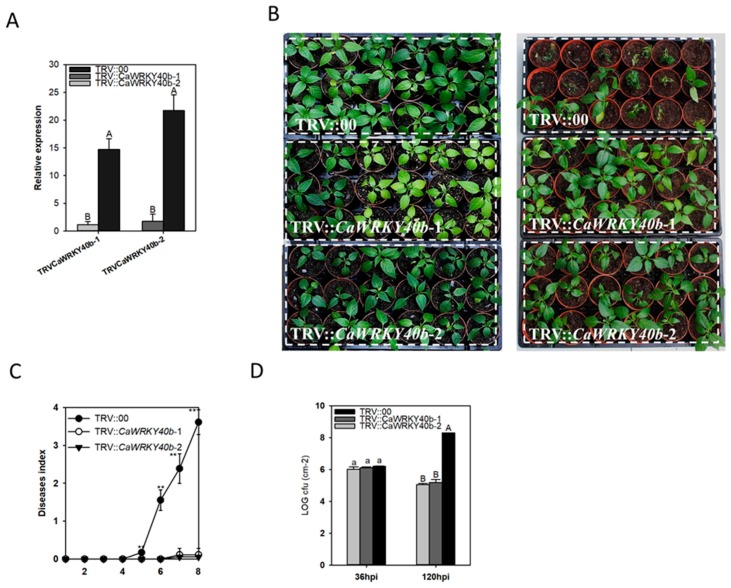
Responses of *CaWRKY40b*-silenced pepper plants to *Ralstonia solanacearum* inoculation. (**A**) Real-time RT-PCR analysis of *CaWRKY40b* expression in leaves of *CaWRKY40b*-silenced pepper plants (TRV::*CaWRKY40b1* and TRV::*CaWRKY40b2*) and control (TRV::00) plants. (**B**) *CaWRKY40b*-silenced pepper plants exhibit similar sizes compared to that in the control plants. Phenotypic effect of *R. solanacearum* inoculation on *CaWRKY40b*-silenced (TRV::*CaWRKY40b1* and TRV::*CaWRKY40b2*) and control (TRV::00) plants at 8 dpi. (**C**) Pepper plants inoculated with *R. solanacearum* were scored every 3 dpi using a disease index ranging 0–4: 0 (no wilting), 1 (1–25% wilted), 2 (26–50% wilted), 3 (51–75% wilted), and 4 (76–100% wilted or dead). (**D**). Detection of growth of *R. solanacearum* in rootstocks of the pathogen-inoculated *CaWRKY40b*-silenced (TRV::*CaWRKY40b1* and TRV::*CaWRKY40b2*) or control pepper plants at 24 hpi and 48 hpi, respectively. For *R. solanacearum* inoculation in (**B**,**C**), all the pots containing pepper plants were placed in a tray containing Hoagland’s nutrient solution supplement with 10 mL of the FJC100301 suspension (OD600 = 0.8) per liter. In (**A**,**C**,**D**), the mean ± SD was calculated from four independent duplicates, with each duplicate consisting of 10 plants. Asterisks in C indicate statistically significant differences compared to the mock treatment by the LSD test (* *p* < 0.05, ** *p* < 0.01). Different letters in (**A**–**D**) indicate significant differences among means as determined by Fisher’s protected LSD test: uppercase letters, *p* < 0.01; lower case letters, *p* < 0.05.

To investigate the possible role of *CaWRKY40b* in the response of pepper to RSI, loss of function analysis was conducted by VIGS in pepper plants. To silence *CaWRKY40b* in pepper plants, two fragments in the 3′ UTR or ORF of *CaWRKY40b* were employed. The specificity of these two fragments was further confirmed by whole-genome search (http://passport.pepper.snu.ac.kr). GV3101 cells containing TRV::*CaWRKY40b* were infiltrated into the leaves of 20-day-old pepper seedlings. The silencing process was monitored by TRV::*PDS* pepper plants. Using real-time RT-PCR, the transcription of *CaWRKY40b* was assayed and after 20 days post inoculation (dpi), the transcript level of *CaWRKY40b* in TRV::*CaWRKY40b-1* and TRV::*CaWRKKY40b-2* pepper plants was <10% of that in the TRV::00 plants (Figure 3A).

The TRV::*CaWRKY40b*-*1*, *CaWRKKY40b-2* and TRV::00 pepper plants were inoculated with *R. solanacearum* cells FJC100301 [4] at 3 dpi. A clear wilting phenotype was observed in the majority of FJC100301-inoculated TRV::00 pepper plants, whereas no obvious or only slight wilting was observed in both TRV::*CaWRKY40b-1* and TRV::*CaWRKY40b-2* pepper plants (Figure 3B). To accurately quantify the extent of disease in *R. solanacearum*-inoculated plants, we determined the relative disease index from 1 dpi to 8 dpi in both the TRV::*CaWRKY40b-1* TRV::*CaWRKY40b-2* and TRV:00 pepper plants. To quantify the growth of *R. solanacearum* in the inoculated pepper rootstocks, the cfu of the rootstocks of *R. solanacearum* inoculated pepper plants was measured. TRV2::*CaWRKY40b-1* and TRV2::*CaWRKY40b-2* consistently showed a significant decrease in disease symptoms as well as pathogen growth, respectively, compared to the wild-type plants (Figure 3C,D).

### 2.5. Transient Overexpression of CaWRKY40b-SRDX Triggered Intensive Cell Death in Pepper Plants

**Figure 4:**
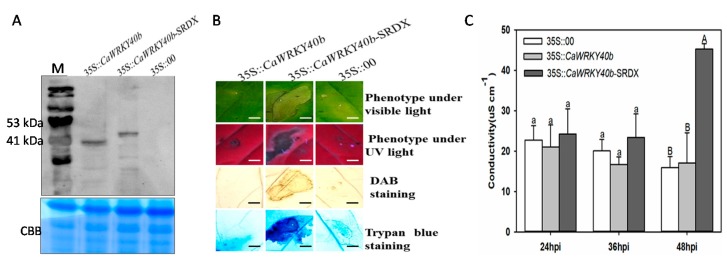
The transient overexpression of *CaWRKY40b*-SRDX triggers extensive HR cell death in pepper leaves. (**A**) The transient overexpression of *CaWRKY40b*-HA-SRDX and *CaWRKY40b*-HA in pepper leaves was detected by immune-blotting (IB) against the antibody of HA. CBB, Coomassie brilliant blue. (**B**) Intensive cell death was triggered by transient overexpression of *CaWRKY40b*-SRDX, but not by that of *CaWRKY40b* and displayed by phenotype, DAB, and trypan blue staining at 4 dpi, respectively. Bar = 100 μm. (**C**) Quantification of electrolyte leakage as ion conductivity to assess the cell death response in leaf disks, the means ± SD were calculated from four samples, each containing six disks. Capital and lowercase letters above the bars indicate significant difference at *p* < 0.01 and *p* < 0.05, respectively, as analyzed by Fisher’s protected LSD test.

The effect of transient overexpression of *CaWRKKY40b* and its chimeric repressor version *CaWRKY40b*-SRDX [46,47] on the hypersensitive response (HR) cell death in leaves of pepper plants was investigated. GV3101 cells containing construct 35S::*CaWRKY40b*, *35S::CaWRKY40b*-SRDX and 35S::00 were infiltrated into the pepper leaves. Transient overexpression of *CaWRKY40b*-HA in pepper leaves was detected by immunoblotting (IB) against the antibody of HA at 48 hpi (Figure 4A). A clear cell death and darker staining of trypan blue and DAB were consistently noticed around infiltrated sites of *Agrobacterium* cells containing *35S::CaWRKY40b-SRDX*; however, no obvious cell death or darker staining of trypan blue or DAB was observed in the *CaWRKY40b*-transiently overexpressing and in the mock-treated pepper leaves (Figure 4B). Furthermore, a higher ion leakage was triggered by *35S::CaWRKY40b-SRDX* than that by *35S::CaWRKY40b* (Figure 4C).

### 2.6. Transcriptional Modulation of Marker Genes by Transient Overexpression and Virus-Induced Silencing of CaWRKY40b

Chromatin Immunoprecipitation sequencing (ChIP-seq) was performed to identify the potential target genes of *CaWRKY40b* (Figure S2). To do this, GV3101 cells harboring *35S::CaWRKY40b-HA* was infiltrated into leaves of six-week-old pepper plants and maintained in the greenhouse. Forty-eight hours post infiltration, the infiltrated leaves were sampled for chromatin isolation, the chromatins were sheared into fragments of 300–500 bps in length and were immunoprecipitated with antibodies of HA, the sequencing of immunoprecipitated DNA was performed on Illumina HiSeq2500 resulted in about one million reads, in which more than 8 hundred binding sites were identified. Gene ontology (GO) was analyzed to determine the biological and functional processes of *CaWRKY40b* target genes (Figure S2 and Table S1). As the role of *CaWRKY40b* in pepper immunity was the focus of the present study, immunity associated genes were selected from the target genes of *CaWRKY40b* to confirm the role of *CaWRKY40b* and to test the possible mode of action, including *CA00g87690* (*CaWRKY40*), *CA05g11520* (*jasmonic acid-amido synthetase JAR1-like isoform X2, JAR1*), *CA06g24540* (*heat shock cognate 70 kDa protein 1, HSC70*), *CA01g13570* (*ETHYLENE INSENSITIVE 3-like, EIN3*), *CA02g12020* (*LRR receptor-like serine/threonine-protein kinase FLS2*), *CA10g01730* (*LRR receptor-like serine/threonine-protein kinase At3g47570 isoform X1, RLK1*), *CA05g11620* (*putative cyclic nucleotide-gated ion channel 8*, *CNGIC8*), and *CA09g10430* (*calcium-dependent protein kinase 13*, *CDPK13*). The result from ChIP-PCR showed that DNA within the promoter regions of these genes enriched in the *CaWRKY40b* bound DNA, attesting the validity of the ChIP-seq results (Table S1 and Figure S3).

**Figure 5:**
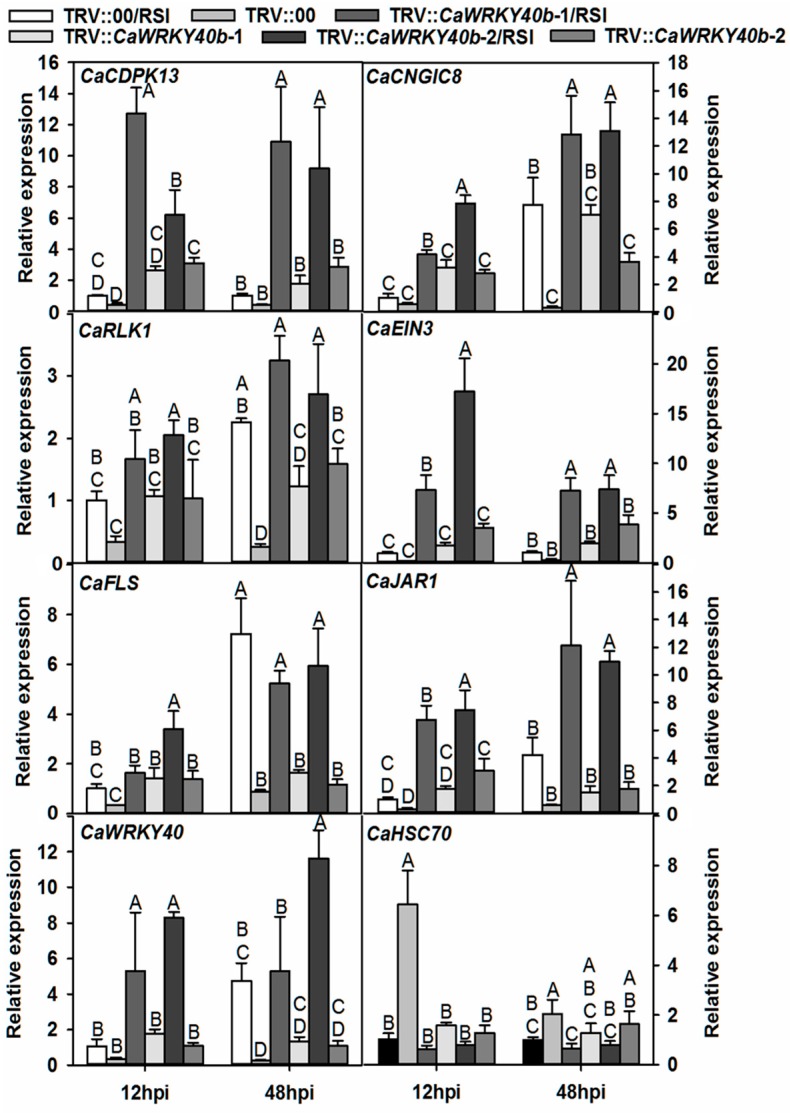
q RT-PCR analyses of transcriptional levels of the tested defense-related genes in the leaves of pepper plants. The *CaWRKY40b*-silenced and the control pepper plants were inoculated with 1.0 mL of 108 cfu·mL^−1^ (OD600 = 0.8) virulent *R. solanacearum* strain FJC100301 by root irrigation. Data represent the means ± SD of four independent biological replicates, each containing three plants. Different capital letters indicate significant differences, as determined by Fisher’s protected LSD test: uppercase letters, *p* < 0.01; lower case letters, *p* < 0.05.

**Figure 6:**
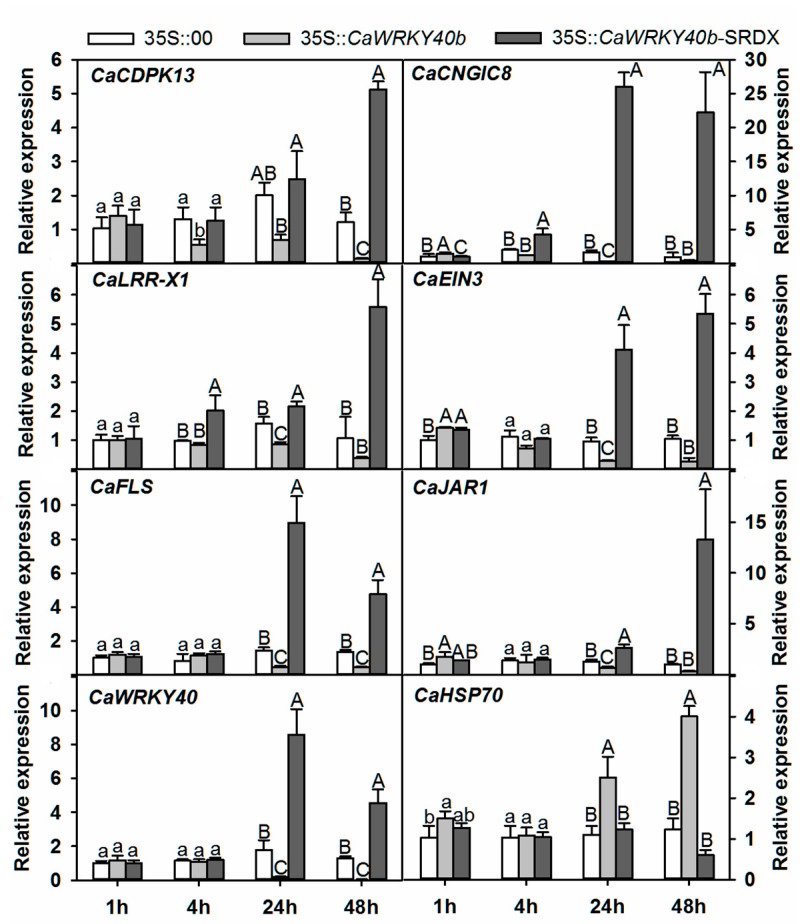
qRT-PCR analysis of the expression of immunity-associated marker genes in *35S::00*, *35S::CaWRKY40b* and *35S::CaWRKY40b-SRDX* constructs. Data display the means ± SD of four independent biological replicates, each containing three leaves. Capital and lowercase letters above the bars indicate significantly different means (*p* < 0.01) and significantly different means (*p* < 0.01), respectively, as analyzed by Fisher’s protected LSD test.

To test whether *CaWRKY40b* can control the transcriptional expression of all the genes directly targeted by CaWRKY40b, their transcription was comparatively tested in *CaWRKY40b* transiently overexpressed and *CaWRKY40b*-silenced pepper plants by real-time RT-PCR. The results demonstrated that the transcription of *CaWRKY40*, *JAR1*, *BPR1*, *EIN3*, *FLS2*, *RLK1*, *CNGIC8*, and *CDPK13* was significantly enhanced by RSI, and the transcription of these genes was significantly higher in *CaWRKY40b*-silenced plants than in the *CaWRKY40b* un-silenced control plants with or without RSI. In contrast, the transcription of *CaHSC70*, which was downregulated by RSI, was lower in *CaWRKY40b*-silenced pepper plants than that in the control plants (Figure 5). Consistent to the result in *CaWRKY40b*-silenced pepper plants, transient overexpression of *CaWRKY40b-SRDX* significantly downregulated *CaHSC70*, while elevated the transcript levels of the other tested genes compared to the control (Figure 6).

### 2.7. The Expression of CaWRKY40b Was Directly and Transcriptionally Regulated by CaWRKY40b Itself

**Figure 7:**
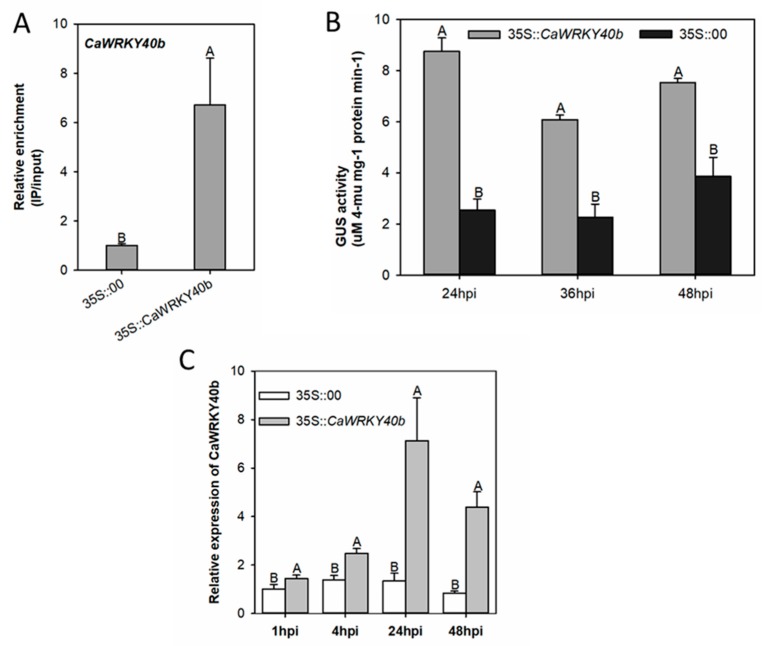
The transcriptional self-regulation of *CaWRKY40b.* (**A**) The binding of *CaWRKY40b* to the promoter of *CaWRKY40b* by ChIP and real-time PCR. GV3101 cells containing *35S::CaWRKY40b*-HA infiltrated into pepper leaves, with GV3101 cells containing 35S::00 as mock treatment. The *CaWRKY40b*-HA overexpressing leaves were harvested at 48 hpi for chromatin preparation. Chromatin was isolated from infiltrated pepper leaves crosslinked with 1% formaldehyde, sheared, and immune-precipitated with an anti-HA antibody. Relative enrichment levels of *CaWRKY40b* in the promoter of *CaWRKY40b* were set to 1 after normalization by input. (**B**) *pCaWRKY40b*-driven GUS expression was triggered by transient overexpression of *CaWRKY40b* after infiltration of *Agrobacterium* into the leaves. The pepper leaves were also co-infiltrated with GV3101 possessing *pCaWRKY40b*::GUS or *35S::CaWRKY40b*. (**C**) The transcript level of *CaWRKY40b* against transient overexpression of *CaWRKY40b* in pepper leaves by real time RT-PCR: for transcript level of *CaWRKY40b* in *CaWRKY40b* overexpressing leaves, the primer pair based on the 3′ UTR was used. Data are the means ± SD of four independent biological replicates. Capital and lowercase letters above the bars indicate significantly different means at *p* < 0.01 and *p* < 0.05, respectively, as analyzed by Fisher’s protected LSD test.

As *CaWRKY40b* itself was identified by ChIP-seq as the target gene of *CaWRKY40b*, the possible binding of *CaWRKY40b* to its own promoter was also confirmed by ChIP-seq (Figure 7A). The possible self-regulation of *CaWRKY40b* was also tested comparatively by assaying the effect of transient overexpression of *CaWRKY40b* on the expression of GUS-driven *pCaWRKY40b* and on the transcription of *CaWRKY40b*. The results presented that the transient overexpression of *CaWRKY40b* significantly increased GUS expression (Figure 7B) as well as the transcriptional level of *CaWRKY40b* by real-time RT-PCR using a pair of specific primers based on the sequence of 3′-UTR of *CaWRKY40b* (Figure 7C).

## 3. Discussion

Data mainly from model plants including *Arabidopsis* and rice suggest that WRKY TFs play important roles in plant immunity, but their role and the underlying mechanism remain poorly understood. Despite functional characterization of several members of this family in pepper [3,4,37,38,40,42,43,48,49], the function of majority of members in this family in pepper remains unknown. The findings of the present study suggest that *CaWRKY40b* acts as a negative regulator in pepper immunity against RSI by directly modulating the transcription of a subset of immunity-associated genes.

WRKY proteins are characterized by their one or two highly conserved *WRKY* domains and bind to the target genes promoters mainly by means of the typical W-box [16,17]. The presence of highly conserved WRKY domains in *CaWRKY40b* and their binding to W-box-containing promoters of their potential target genes as indicated by ChIP assay and real-time PCR suggest that *CaWRKY40b* is a member of the WRKY family of pepper. In addition, our results indicate that *CaWRKY40b* acts as a negative regulator in the response of pepper to *R. solanacearum*, and silencing of *CaWRKY40b* with two independent VIGS vectors significantly and consistently decreased the growth of *R. solanacearum* and the susceptibility of pepper plants to RSI. Similarly, transient overexpression of *CaWRKY40* chimeric repressor version (e.g., *CaWRKY40*-SRDX) [46,47] triggered intensive HR cell death in pepper leaves whereas that of *CaWRKY40b* did not. The role of *CaWRKY40b* as a negative regulator in pepper immunity is similar to that of its homolog, *AtWRKY40*, in *Arabidopsis* [34], but different from its ortholog *CaWRKY40* in pepper [4]. Further investigation into how this functional difference between *CaWRKY40* and *CaWRKY40b* is structurally determined would provide new insights into pepper immunity. Because *CaWRKY40b* was downregulated by RSI, we hypothesize that the constitutive expression of *CaWRKY40b* blocks defense responses to minimize the unnecessary resource cost for defense responses in the absence of pathogens, whereas its downregulation might derepress immunity with RSI challenge. Besides *CaWRKY40b*, *CaWRKY1* [37] and *CaWRKY58* [44] have been characterized in pepper as negative regulators in the immunity [44,50]. Gene silencing of *CaWRKY40b* in this study or *CaWRKY1* and *CaWRKY58* in previous studies [37,44] significantly decreased the susceptibility of pepper to pathogen attack, thereby suggesting their functional specificities. The existence of multiple negative regulators might favor plants to avert inappropriate activation of different defense responses or activate defense responses against pathogens with different lifestyles.

The role of *CaWRKY40b* as a negative regulator in pepper immunity was further supported by its direct modulation of the transcription of a subset of immunity-associated genes. These genes originally identified by ChIP-seq, including *CaWRKY40*, *JAR*, *HSC70*, *RLK1*, *EIN3*, *FLS2*, *CNGIC8*, and *CDPK13*, which act as positive regulators in plant immunity, were downregulated by transient overexpression of *CaWRKY40b*, but upregulated by silencing of *CaWRKY40b*. Although the exact roles of the tested target genes in pepper immunity remain to be identified, *JAR* [51,52,53] and *EIN3* [54,55] have been implicated in JA and ET signaling pathways, which ubiquitously exist in the immune system of different plant species. *RLK1* [56,57] and *FLS2* [58,59,60,61], which are PRRs crucial for perception of conserved MAMPs to activate PTI, are conserved among different plant species [5,6]. Most importantly, our previous study provided concrete evidence that *CaWRKY40* acts as positive regulator in the response of pepper to RSI [3,4]. Therefore, we hypothesize that *CaWRKY40b* functions as a negative regulator in pepper immunity at least partially by repressing these positive regulators. In contrast, *HSC70*, a negative regulator in plant immunity [62] was downregulated by *CaWRKY40b* silencing but enhanced by overexpression of *CaWRKY40b*, indicating that *CaWRKY40b* also acts as a negative regulator by activating negative regulators, and *CaWRKY40b* possesses dual functionality, acting either as a repressor or as an activator in a promoter-context dependent manner, similar to *AtWRKY33* [63]. In addition to these target genes, *CaNPR1* [3,4,64,65,66] and *CaDEF1* [67,68,69], which were found to be non-target genes of *CaWRKY40b*, were also consistently upregulated by *CaWRKY40b* silencing and downregulated by transient overexpression of *CaWRKY40b*. All these findings support the role of *CaWRKY40b* as a negative regulator that directly regulates immunity at multiple levels, including signaling regulatory proteins, TFs, and PR proteins.

WRKY genes have been suggested to be functionally connected by forming transcriptional networks [35,36]. Our data in the present study illustrate that *CaWRKY40*, which was previously found to be transcriptionally modulated directly by *CaWRKY6* [3], is directly targeted and transcriptionally regulated by *CaWRKY40b*. Additionally, typical W-boxes were also found to be enriched in the promoters of *CaWRKY6* and *CaWRKY40*, as well as the promoter of *CaWRKY40b* (Figure 1A), indicating the participation of these *WRKYs* in a *WRKY* web during the regulation of pepper responses to *R. solanacearum*. The present study also revealed that *CaWRKY40b* positively modulates the transcription of *CaWRKY40b* by directly binding to the promoter of *CaWRKY40b*. A similar positive feedback regulation has been frequently reported in the *WRKY* network [35,70].

In sum, *CaWRKY40b* acts as a negative regulator of pepper resistance to RSI by transcriptionally regulating a set of immunity-associated genes, including *CaWRKY40.* Its downregulation upon RSI derepresses immunity, but its upregulation in healthy plants impairs pepper immunity.

## 4. Materials and Methods

### 4.1. Plant Materials and Growth Conditions

Seeds of pepper (*C. annuum*) inbred line 68-2, which has an intermediate level of resistance to *R. solanacearum*, and *Nicotiana benthamiana* were obtained from the pepper breeding group at the Fujian Agriculture and Forestry University (Fuzhou, China). Seeds were sown in in plastic pots containing soil with a peat moss:perlite ratio of (2:1, *v*/*v*) at 25 ± 2 °C, 70% relative humidity, 60–70 μmol photons·m^−2^·s^−1^, and 16 h light/8 h dark [71,72].

### 4.2. R. solanacearum Inoculation

A virulent *R. solanacearum* strain FJC100301 was isolated from wilted pepper samples at our laboratory in Fujian Province (China) and amplified as described elsewhere [4]. The *R. solanacearum* strain was cultured in PSA medium (200 g/L of potato, 20 g/L of sucrose, 3 g/L of beef extract, and 5 g/L of tryptone) at 28 ± 2 °C and 200 rpm and then re-suspended in a 10 mM MgCl_2_ solution. The bacterial cell solution used for inoculation was diluted to 10^8^ cfu·mL^−1^ (OD600 = 0.8). To inoculate pepper plants with *R. solanacearum* by root irrigation, pepper plants grown in pots were placed in a tray containing Hoagland’s nutrient solution supplemented with the FJC100301 suspension. To inoculate pepper leaves with *R. solanacearum*, 10 μL of the *R. solanacearum* suspension (OD600 = 0.8) was infiltrated into the top third leaves of pepper plants at the eight-leaf stage using a needleless syringe, whereas pepper plants inoculated with 10 mM MgCl_2_ was used as mock control.

### 4.3. Vector Construction

To construct vectors for overexpression, subcellular localization, and ChIP assay, the full-length ORF of *CaWRKY40b* (with or without a termination codon) was cloned into the entry vector pDONR207 by BP reaction. This entry vector was then cloned into destination vectors pMDC83 and 3687-HA by LR reaction using the Gateway^®^ cloning technique (Invitrogen, Carlsbad, CA, USA). In addition, the EAR repression domain (SRDX) was fused to the 3′ terminus of the ORF of *CaWRKY40b*; the resulting *CaWRKY40b*-SRDX was cloned into pEarleyGate201 as previously described. To construct the vector for VIGS, a 229-bp fragment in 3′-untranslated region (UTR) of *CaWRKY40b* and a fragment in its ORF were selected for vector construction, and their specificities were confirmed by BLAST against the genome sequence in the databases of CM334 (http://peppergenome.snu.ac.kr/) and Zunla-1 (http://peppersequence.genomics.cn/page/species/blast.jsp). The specific fragment was cloned into the entry vector pDONR207, and then cloned into the PYL279 vector.

### 4.4. Subcellular Localization

*Agrobacterium tumefaciens* strain GV3101 containing the constructs *35S::CaWRKY40b-GFP* or *35S::GFP* (used as a control) were grown overnight, respectively, and then resuspended in the induction medium. Bacterial suspensions (OD600 = 0.8) were injected into *N. benthamiana* leaves using a syringe without a needle. At 48 hpi, GFP fluorescence was imaged using a Laser Scanning Confocal Microscope (TCS SP8, Leica, Solms, Germany) at an excitation wavelength of 488 nm and a 505–530 nm band-pass emission filter.

### 4.5. VIGS of CaWRKY40b in Pepper Plants

For VIGS of *CaWRKY40b* in 68-2 line pepper plants, *A. tumefaciens* strain GV3101 harboring PYL192, PYL 279-*CaWRKY40b1* and PYL279-*CaWRKY40b2* or PYL279 (resuspended in the induction medium at a 1:1 ratio, OD600 = 0.6) were co-infiltrated into the cotyledons of two-week-old pepper plants. The detailed process was conducted according to our previous studies [3,4].

### 4.6. Transient Overexpression of CaWRKY40b in Pepper Leaves

For transient overexpression analysis, *A. tumefaciens* strain GV3101 harboring the *35S::CaWRKY40b* or *35S::CaWRKY40b-HA*, *35S::CaWRKY40b-SRDX* or *35S::00* vector (the empty vector was used as control) was both shaking-grown overnight. The *agrobacterium* pellets were collected by centrifugation and resuspended in the induction medium (10 mM MES, 10 mM MgCl_2_, 200 µM acetosyringone, pH 5.6). The adjusted bacterial suspension (OD600 = 0.8) was vacuum-infiltrated into the leaves of 6-week-old pepper plants using a needleless syringe and the injected leaves were maintained in the greenhouse. The samples were collected at the indicated time points for further analysis.

### 4.7. Histochemical Staining

Staining of the leaves with trypan blue and diaminobenzidine (DAB) was used as described elsewhere [73] and in our previous studies [3,74].

### 4.8. Quantitative Real-Time RT-PCR

To determine the relative transcript accumulations of target genes, real-time PCR was performed using specific primers (Table S2) according to manuals of BIO-RAD real-time PCR system (Foster City, CA, USA) and SYBR Premix Ex Taq II system (TaKaRa). Total RNA extraction and real-time RT-PCR were performed as earlier described [3,74]. Four independent biological replicates of each treatment were performed. The Livak method [75] was used to analyze the real-time PCR data. The data were expressed as a normalized relative expression level (2^−ΔΔ*C*t^) of the respective genes. The transcript accumulation of *CaActin* (GQ339766) and *18S ribosomal RNA* (EF564281) were, respectively, used to normalize the relative transcriptional level of each sample. 

### 4.9. ChIP Analysis

ChIP assays were performed by following a previously described protocol with slight modifications [76]. Three to four fully expanded leaves of plants at the eight-leaf stage were inoculated with GV3101 cells containing *35S:CaWKRY40b-HA* or *35S:00* (used as a control). The inoculated leaves were collected at 24 hpi; about 4 g of leaves were treated with 1.0% formaldehyde for 8 min, to which 3 M glycine was added to a final concentration of 0.125 M. The sample was then vacuumed for 5 min to stop cross linking. Nuclear extracts were isolated and were resuspended with the extraction buffer I (0.4 M sucrose, 10 mM Tris-HCl, pH 8.0, 10 mM MgCl_2_, 5 mM β-mercaptoethanol, 1 U protease Inhibitors), II (0.25 M sucrose, 10 mM Tris-Cl, pH 8.0, 10 mM MgCl_2_, 1% Triton X-100, 5 mM β-mercaptoethanol, 1 µL protease inhibitors), and III (1.7 M sucrose, 10 mM Tris-HCl, pH 8.0, 2 mM MgCl_2_, 0.15% Triton X-100, 5 mM β-mercaptoethanol, 1 µL protease Inhibitors) sequentially, and then digested with micrococcal nuclease (Takara, Dalian, China), according to the manufacturer’s instructions. Magnetic beads (Invitrogen, Carlsbad, CA, USA) linked to the antibody of HA (anti-HA tag rabbit polyclonal antibody, Sigma, St. Louis, MO, USA) were added to the digested samples, and then eluted. Later, the protein–DNA complex was digested with 2 mL of 10 mg·mL^−1^ proteinase K and incubated at dry bath with 45 °C for 1 h and same volume of Tris-saturated phenol:chloroform:isoamyl alcohol (25:24:1 *v*/*v*) was used to extract the DNA solution twice. Then, DNA was precipitated by adding 3 mL of 100% ethanol, 1/10 volume of 3 M NaOAc, and 1 mL of 2 M glycogen along with overnight incubation at −20 °C. DNA was pelleted by spinning for 20 min at 16,700× *g*. The DNA pellets were washed with 80% ethanol, dried at room temperature, resuspended in 50 μL TE buffer, and stored at −20 °C until use for ChIP-seq and ChIP-PCR. For ChIP-seq, the immunoprecipited DNA samples were used to generate sequencing libraries bearing barcodes using a NEBNext ChIP-seq Library PreReagent Set for Illumina kit (New England Biolabs, Ipswich, MA, USA). Sequencing was performed on Illumina HiSeq2500 at Nevogene (Beijing, China). ChIP-seq data analysis was performed following the method used by Liu et al. [63]. Quantitative real-time PCR was used to analyze the immune-precipitated DNA for enrichment of *CaWRKY40b* at the promoter region of the target genes. Fold increases of immune-precipitated DNA were calculated relative to the input DNA and the internal control *CaACTIN* or *18S rRNA*. ChIP-PCR was performed at least in triplicate.

### 4.10. Fluorometric GUS Enzymatic Assay

A fluorometric GUS enzymatic assay for measuring GUS activity in pepper plant extracts was performed by adopting a previously described protocol [4].

### 4.11. Immunoblotting

Total protein extracts were incubated with anti-HA agarose (Thermo Fisher Scientific, Waltham, MA, USA) overnight at 4 °C. Beads were collected and washed with Tris-buffered saline and Tween-20 (0.05%). Eluted proteins were analyzed by immune-blotting using an anti-HA–peroxidase antibody (Abcam, Cambridge, UK).

### 4.12. Quantification of R. solanacearum Growth in Pepper Plants

The growth of *R. solanacearum* was quantified by measuring the colony-forming units (cfu), and the rootstocks of *R. solanacearum*-inoculated pepper plants were harvested at indicated time points, which were ground into powder in liquid nitrogen. For every 1.0 g of powder, 1.0 mL of 10 mM MgCl_2_ solution was added and spun down, the supernatant was collected and diluted into 10,000 times, and 1 μL of the supernatant was added to the PSA plate, which was kept at 28 °C. Approximately 48 h later, the cfus were calculated.

## Supplementary Materials

Supplementary materials can be found at http://www.mdpi.com/1422-0067/19/5/1403/s1.
