# Supplementary material for: CaWRKY40b in Pepper Acts as a Negative Regulator in Response to Ralstonia solanacearum by Directly Modulating Defense Genes Including CaWRKY40
Source: Int J Mol Sci. 2018 May 8;19(5):1403. doi: 10.3390/ijms19051403 (PMC5983674; doi:10.3390/ijms19051403)
Supplement: Supplementary file 1 [file ijms-19-01403-s001.zip › Supplementary figures.pdf]

A

MEFTSLVDTSLDLSFRPLRVPDDIPKQEVESNFIGLGRDLMPDKDDQAGDLLEELNRVSAENKKLTEMLTVMCQNYN

ALRNQLTEYLNK NSTTSTAADNNHDHHSDGSKKRKVENNNNEIVKSVQGLHSESSSSDEDSSNKKPREQHIKTNT

CRVYVKTEASDTSLIVKDGYQ**WRKYGQK**VTRDNPSPRAYFKCSFAPTC PVKKKVQ RSVEDQSILVATYEGE**H**N**H**SK

MDGSGPVTTSPSSRLNPKNTLVGANTTTVMPCSSTSIINTPSGPTLTLDLTQPKKLQNDQKKVNSNTSTSNASGQKSK

SPGGHDHHQQNRPEFQQLFIDQMASSLTKDPSFQAALAAAISGKFLQNNHTDK*

B

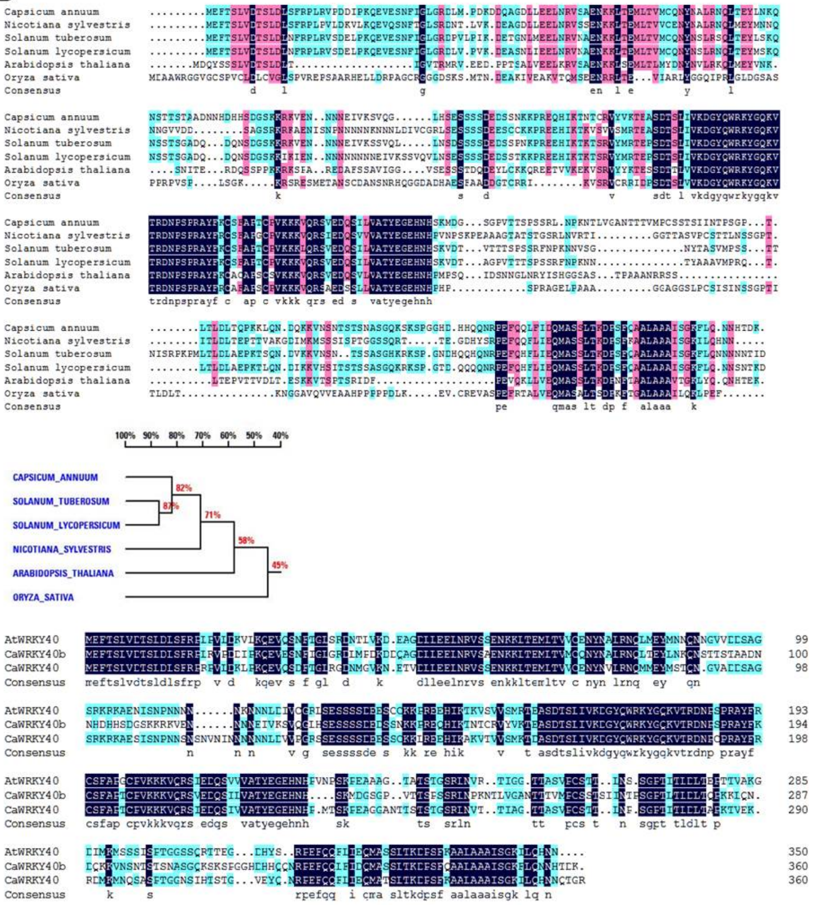

Figure S1. The sequence analysis of CaWRKY40b indicate it is a member of subgroup IIa WRKY. (A) Predicted primary structure of the *CaWRKY40b* based on the cloned cDNA sequence. The WRKY DNA-binding domain is indicated by a gray background. Similar to most of the WRKY domains, our reported 'WRKYGQK' motif is also invariant along with cysteine and histidine residues of the zinc-finger motif that is conserved in WRKY domains. The bold dark grey background (Fig. S1) represents the putative nuclear localization signal. A type II WRKY-specific coiled-coil motif identified by "coils" (http://www.ch.embnet.org/software/COILS_form.html) at the N-termini is underlined. (B) Comparison of amino acid sequences of *CaWRKY40b* with the representative closely related proteins and its homologues in *Solanum lycopersicum* (XP_006341684.1), *S. tuberosum* (XP_006356251.1), *N. sylvestris* (XP_009802478.1), *A. thaliana* (NP_178199.1), and *O. sativa* (Q6IEK5.1). Black shading indicates 100% identity, red shading indicates 75% to 100% identity,

and green shading indicates 50% to 75% identity. Alignment was conducted by the DNAMAN5. *CaWRKY40b* has 59.46% sequence identity with *CaWRKY40.*

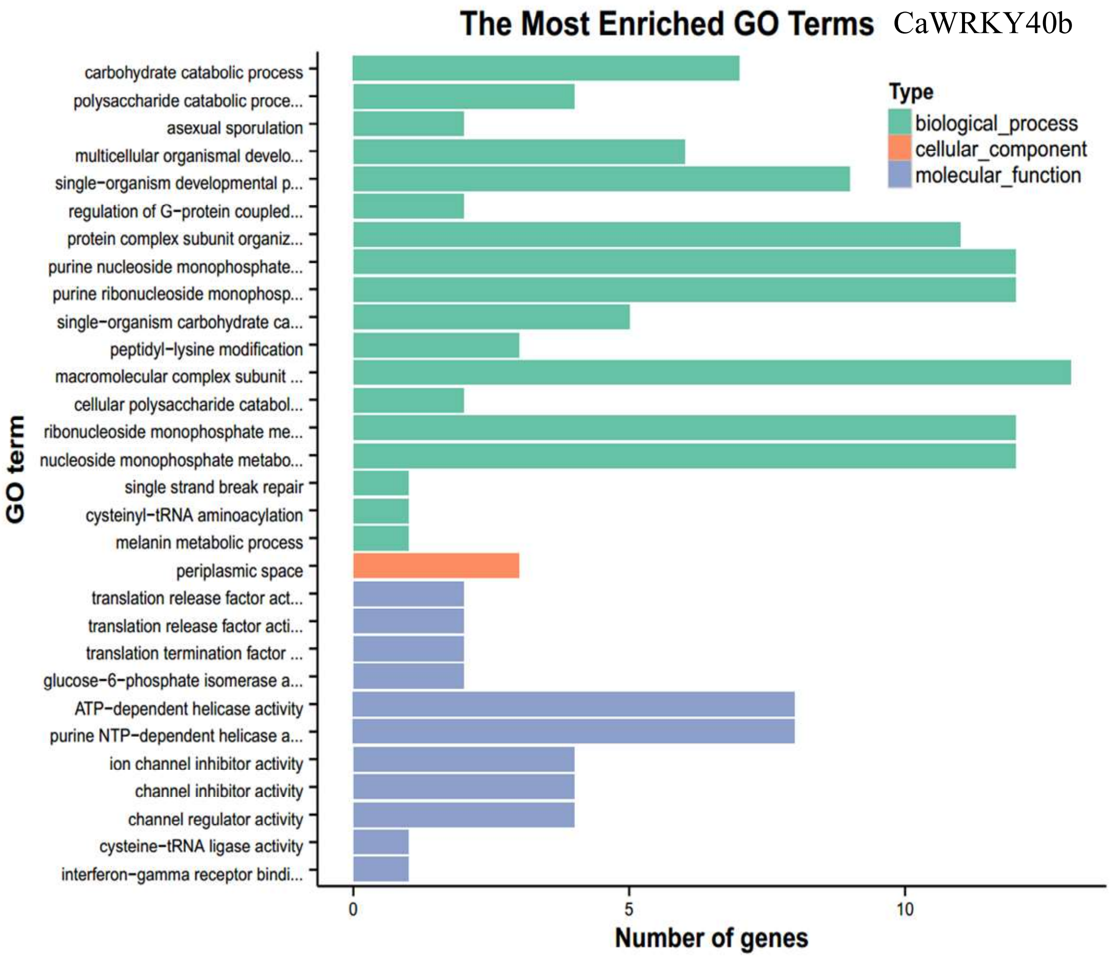

Figure S2. The gene ontology analysis of the direct transcriptional target genes of CaWRKY40b from ChIP-seq.

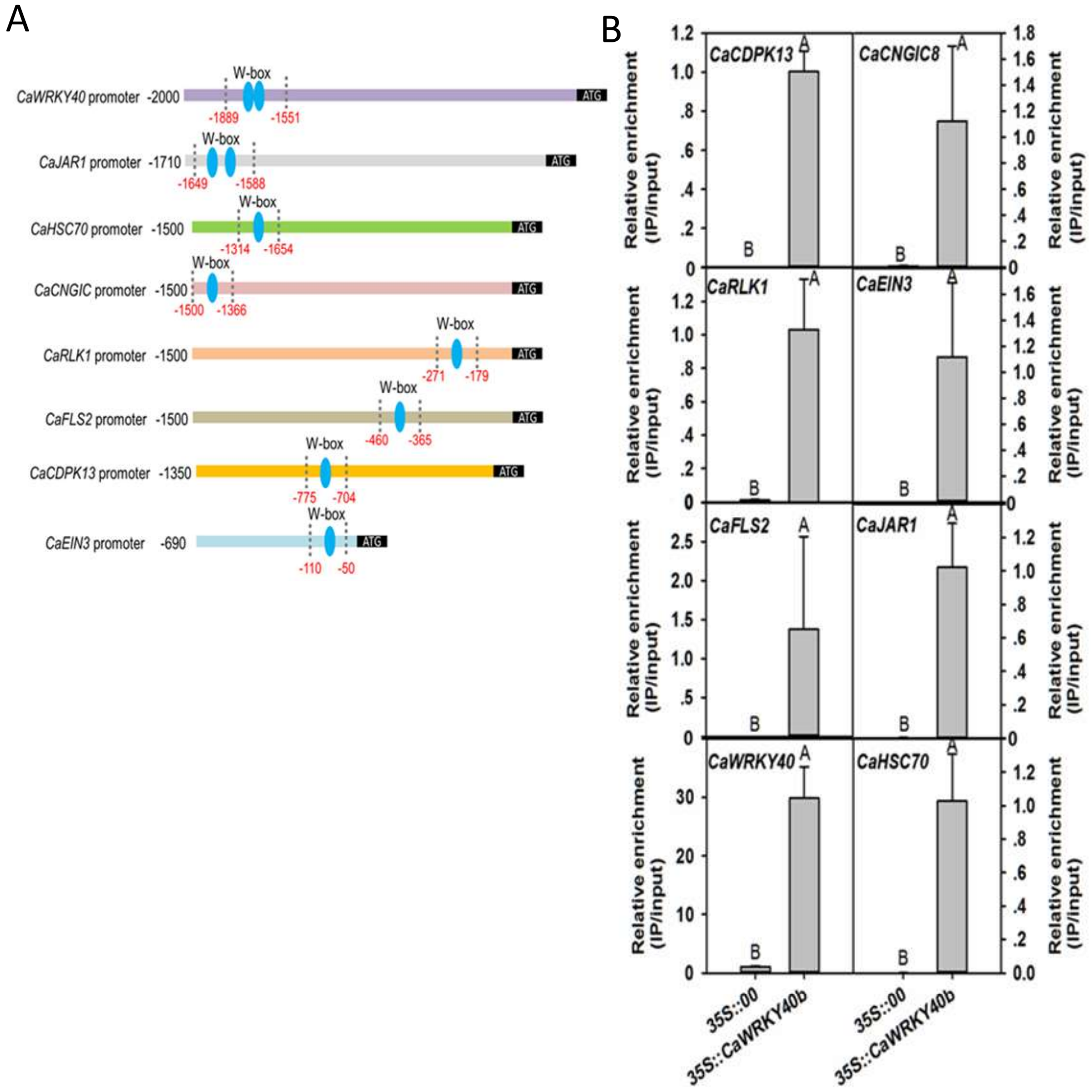

Figure S3. Confirmation of the specific binding between *CaWRKY40b* and the promoters of its possible target genes identified by ChIP-seq and RT-PCR. (A) *CaWRKY40b* binds to the promoters of *CaWRKY40* and *CaJAR1* harboring two W boxes each at their binding sites, *CaWRKY40b* also binds to the promoters of *CaHSC70*, *CaCNGIC8*, *CaRLK1*, *CaFLS2*, *CaCDP13*, and *CaEIN3* that possess one W box each at their binding sites. (B) GV3101 cells containing 35S::*CaWRKY40b*-HA infiltrated into pepper leaves, with GV3101 cells containing 35S::00 as mock treatment. The *CaWRKY40b*-HA overexpressed and mock leaves were harvested at 48 hpi for chromatin preparation. Chromatin was isolated from infiltrated pepper leaves crosslinked with 1% formaldehyde, sheared, and immune-precipitated with an anti-HA antibody. Relative enrichment levels of samples of *CaWRKY40b* were set to 1, normalized by input. Data represent the means ± SD of three independent biological replicates. Capital and lowercase letters above the bars indicate significantly different means (p < 0.01) and significantly different means (p < 0.05), respectively, as analyzed by Fisher's protected LSD test.
